# Expression of Immune Related Genes and Possible Regulatory Mechanisms in Alzheimer’s Disease

**DOI:** 10.3389/fimmu.2021.768966

**Published:** 2021-11-05

**Authors:** Yanjun Lu, Ke Li, Yu Hu, Xiong Wang

**Affiliations:** ^1^ Department of Laboratory Medicine, Tongji Hospital, Tongji Medical College, Huazhong University of Science and Technology, Wuhan, China; ^2^ Department of Blood Transfusion, Tongji Hospital, Tongji Medical College, Huazhong University of Science and Technology, Wuhan, China; ^3^ Institute of Pathology, Tongji Hospital, Tongji Medical College, Huazhong University of Science and Technology, Wuhan, China

**Keywords:** Alzheimer’s disease, immune infiltration, NK cells, single-cell sequencing, immunity related genes

## Abstract

Immune infiltration of peripheral natural killer (NK) cells in the brain has been observed in Alzheimer’s disease (AD). Immunity-related genes (IRGs) play an essential role in immune infiltration; however, the expression of IRGs and possible regulatory mechanisms involved in AD remain unclear. The peripheral blood mononuclear cells (PBMCs) single-cell RNA (scRNA) sequencing data from patients with AD were analyzed and PBMCs obtained from the ImmPort database were screened for cluster marker genes. IRG activity was calculated using the AUCell package. A bulk sequencing dataset of AD brain tissues was analyzed to explore common IRGs between PBMCs and the brain. Relevant regulatory transcription factors (TFs) were identified from the Human TFDB database. The protein-protein interaction network of key TFs were generated using the STRING database. Eight clusters were identified, including memory CD4 T, NKT, NK, B, DC, CD8 T cells, and platelets. NK cells were significantly decreased in patients with AD, while CD4 T cells were increased. NK and DC cells exhibited the highest IRG activity. GO and KEGG analyses of the scRNA and bulk sequencing data showed that the DEGs focused on the immune response. Seventy common IRGs were found in both peripheral NK cells and the brain. Seventeen TFs were associated with IRG expression, and the PPI network indicated that STAT3, IRF1, and REL were the hub TFs. In conclusion, we propose that peripheral NK cells may infiltrate the brain and contribute to neuroinflammatory changes in AD through bioinformatic analysis of scRNA and bulk sequencing data. Moreover, STAT3 may be involved in the transcriptional regulation of IRGs in NK cells.

## Introduction

Alzheimer’s disease (AD) is the most common form of progressive dementia and is characterized by memory decline and severe disability. Pathologically, AD brains harbor neurofibrillary tangles (NFTs) formed by excessive intracellular hyperphosphorylation and misfolded tau and amyloid plaques of extracellular amyloid β (Aβ) (1). In recent years, neuroinflammation and tissue-resident immune cells have been increasingly recognized as critical contributors to AD pathogenesis (2, 3).

Brain-resident microglia are the main component of the local immune system in the central nervous system (CNS), and peripheral immune cells are absent or rare in a healthy brain. Additionally, the blood-brain barrier (BBB) establishes immune privilege for the CNS immune system (4). However, infiltration of peripheral immune cells has been observed in both AD mouse models and human AD brains (5, 6). Recent studies have supported the pathogenic role of circulating immune cells in AD brains. Neutrophil depletion or suppression of neutrophil trafficking ameliorated memory impairment in 3xTg-AD transgenic mice (5). Depletion of NK cells by anti-NK1.1 antibody enhanced neurogenesis, reduced neuroinflammation, and ameliorated cognitive impairment in 3xTg-AD mice (7). These studies have confirmed the interaction between peripheral immune cells and the CNS in AD. Immunity-related genes (IRGs) play essential roles in immune infiltration; however, the expression characteristics of IRGs and possible regulatory mechanisms of immune infiltration in AD remain unclear.

We investigated the expression characteristics of IRGs and possible regulatory mechanisms in AD through bioinformatic analysis combining single-cell RNA (scRNA) and bulk sequencing data.

## Methods

### scRNA Sequencing Data Processing

CD45+ peripheral blood mononuclear cells (PBMCs) were sorted with MoFlo XDP (Beckman Coulter) and subjected to scRNA sequencing using an Illumina NovaSeq 6000 (8). The clean data (GSE181279) alignment to the human reference genome (hg19) were processed with Cell Ranger (version3.0.2, 10X Genomics) to generate the feature-barcode gene expression matrix. Seurat R package (version 4.0.2) was used for downstream principal component analysis (PCA) and t-distributed stochastic neighbor embedding (t-SNE) analysis (9). Cells with <200 genes, >2,500 genes, or >5% mitochondrial genes were filtered out. A total of 24,679 filtered cells were selected for analysis. Gene expression was normalized using the “LogNormalize” method and further scaled. After data normalization, 2000 highly variable genes (HVGs) were identified with “vst” method for each sample. Subsequently, PCA was applied to identify significant principal components (PCs), and the P-value distribution was visualized using the JackStraw and ScoreJackStraw functions. The “Harmony” R package (version 0.1.0) was used for batch correction (10) to avoid the batch effect of sample identity which might disrupt the downstream analysis. Finally, ten PCs were selected for t-SNE analysis. The FindClusters function was used to classify the cells into eight different clusters with a resolution of 0.2. The FindAllMarkers function with logfc.threshold = 0.25 was applied to identify differentially expressed genes (DEGs) for each cluster. Cell type identification was performed based on the DEGs in each cluster and manually checked according to a previous study (11).

For the GSE142853 scRNA sequencing dataset, 3,250 cells were selected. The FindClusters function was used with a parameter resolution of 0.5. The remaining data processing method was the same as that of GSE181279.

### IRGs Score

DEGs of each cluster were screened to identify IRGs based on the ImmPort database (https://www.immport.org/shared/home) (12), and 212 IRGs within the DEGs were selected for IRGs scoring with the AUCell (Version 1.12.0) (13). The AUCell R package scores pathways for each cell were based on gene set enrichment analysis. According to the area under the curve (AUC) value of the selected 212 IRGs, gene expression rankings of each cell were generated to estimate the highly expressed gene set proportion in each cell. Cells expressing more genes within the gene set had higher AUC values. The “AUCell_exploreThresholds” function was used to determine the threshold to identify gene set active cells. Then, the AUC score of each cell was mapped to the UMAP embedding using the ggplot2 R package (Version 3.3.5) to visualize the active clusters.

### Bulk Sequencing Data Processing

Raw data of GSE33000 was downloaded from the GEO database using the GEOquery package (Version 2.58.0) (14). DEGs were calculated using the limma package (version 3.46.0) (15). DEGs with an adjusted P value <0.05, and an absolute logFC > 0.03 were considered to be significantly dysregulated genes. Volcano and heatmap plots were drawn using the ggplot2 package (version 3.3.5). All datasets enrolled in this study are listed in detail in **Table 1**.

**Table 1:** The enrolled datasets in the current study.

Datasets	Type	Platform	Sample size (Control/AD)	Cells (Control/AD)
GSE181279	scRNA sequencing	GPL24676 Illumina NovaSeq 6000 (Homo sapiens)	2/3	10,646/14,033
GSE142853	scRNA sequencing	GPL19057 Illumina NextSeq 500 (Mus musculus)	1/1	1,616/1,634
GSE33000	Microarray	GPL4372 Rosetta/Merck Human 44k 1.1 microarray	157/310	–

### GO and KEGG Analysis

The DEGs for the NK cluster in GSE181279 and DEGs in GSE33000 were independently imported into Enrichr, an online bioinformatic website (https://maayanlab.cloud/Enrichr/) for Gene Ontology (GO) and Kyoto Encyclopedia of Genes and Genomes (KEGG) analysis (16). The top 10 pathways were selected based on p-value ranking.

### PPI Network Construction

Protein-protein interaction (PPI) network analysis was performed using STRING (https://string-db.org/) (17).

## Results

### scRNA Profiling of PBMCs in AD

**Figure 1:**
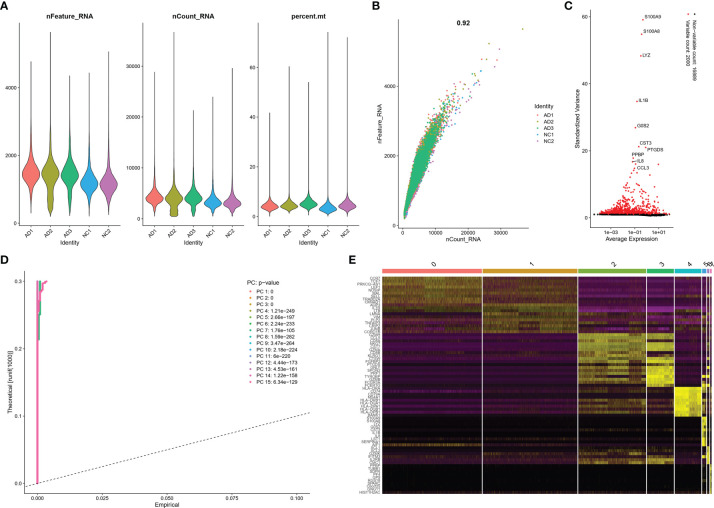
scRNA analysis of PBMCs in AD. **(A)** The genes (features), counts, and mitochondrial gene percentage of each sample. **(B)** Correlation between genes and counts in each sample. **(C)** HVGs were colored in red, and the top 10 HVGs were labeled. **(D)** PCs selection using JackStraw function. **(E)** Heatmap of top 10 DEGs in each cluster. The top 10 DEGs were labeled in yellow color.

The scRNA sequencing dataset (GSE181279) from the GEO database was analyzed, which included 36,849 PBMCs, comprising 22,775 cells from AD patients and 14,074 cells from controls (NC). After filtration, 24,679 cells comprising 14,033 cells from AD patients and 10,646 cells from NC were retained. The expression characteristics of each sample are shown in **Figure 1A**). The nCount_RNA, which represents the number of unique molecular identifiers (UMI), positively correlated with nFeature_RNA, which represented the number of genes, with a correlation coefficient of 0.92 (**Figure 1B**). The top 10 HVGs were identified (**Figure 1C**). S100A8 and S100A9 are the top two HVGs, which are small calcium-binding proteins that are highly expressed during inflammatory conditions (18). PCA identified all 15 PCs with P value <0.05, as visualized with JackStrawPlot (**Figure 1D**), eight clusters were identified using 10 PCs, and the top 10 DEGs of each cluster are listed (**Figure 1E**).

**Figure 2:**
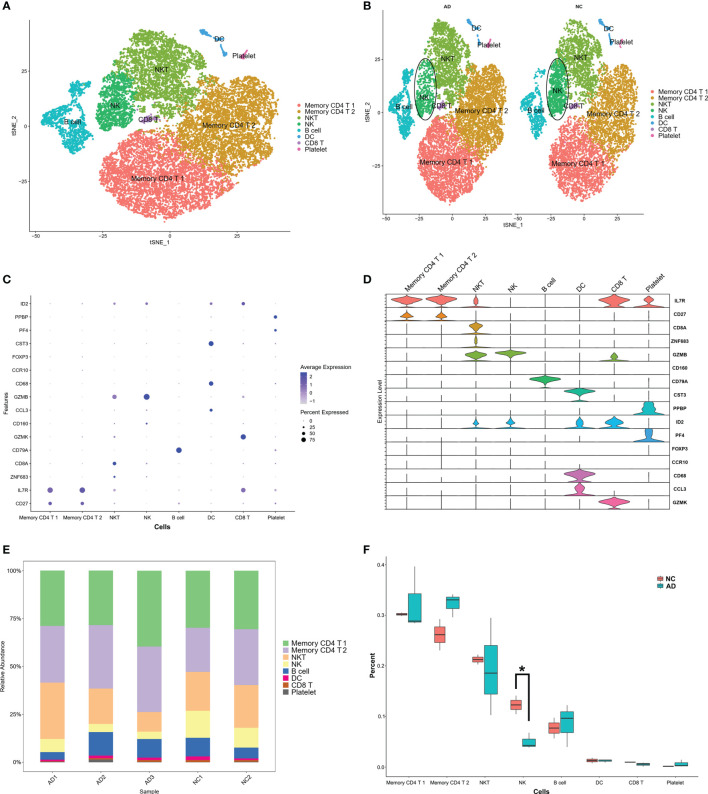
Marker gene expression of each cluster. **(A)** tSNE projection of 24,679 cells from all PBMCs. Different cell types were colored with unique colors. **(B)** tSNE projection of AD and NC groups, respectively. NK cells were labeled with ellipse tag, which were decreased in AD group. **(C)** Dot plot of cell type marker genes. Cell specific marker genes were selected according to previous study by Sinha D (11). The color of dots represents average expression, and size of dots represents average percent of cells expressing selected gene. **(D)** Violin plot depicts distributions of cell type marker genes in each cluster using density curves. The width of each violin plot corresponds with the frequency of cells with relevant gene expression level. **(E)** Cluster distribution in each sample. **(F)** Cluster distribution in AD and NC group. The percent of NK cluster was decreased in PBMC of AD group. * means, P < 0.05 compared with NC group.

These clusters could be assigned to known cell lineages through marker genes, according to a previous study (11). The eight clusters were visualized using the t-SNE analysis (**Figure 2A** and **Table S1**). The ellipse-tagged NK cluster revealed a decreased percentage of NK clusters in the AD group (**Figure 2B**). The expression of cell type marker genes is shown in the dot plot (**Figure 2C**) and Violin plot (**Figure 2D**). The number and proportion of cells in each cluster are shown in **Figure 2E** and **Table 2**, respectively. The NK cluster was significantly reduced in the AD group compared with the NC group (5% vs. 21%), while the CD4 T cells showed an increasing trend in the AD group (**Figure 2F**). In the subsequent analysis, we mainly focused on the NK cluster of the PBMCs.

**Table 2:** Cell numbers of each cluster.

Cluster	0	1	2	3	4	5	6	7
AD1	1568	1609	1603	369	216	49	6	15
AD2	1496	1739	977	225	644	74	31	76
AD3	1323	1139	343	129	322	46	29	5
NC1	1823	1408	1237	862	596	112	63	5
NC2	1386	1328	1009	475	257	34	41	10

### IRG Scores of PBMC Cell Clusters in AD

**Figure 3:**
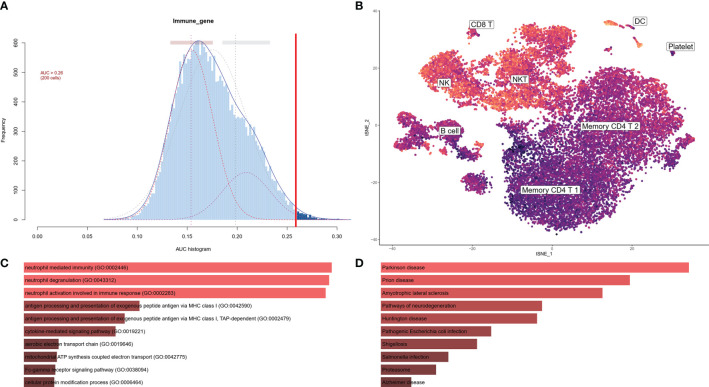
IRG scores of PBMC cell clusters in AD. **(A)** Score of 212 screened IRGs. The threshold was chosen as 0.26. **(B)** t-SNE plots of IRG score in all clusters. NK and DC cells express more genes and exhibit higher AUC values. **(C)** GO analysis of DEGs in NK cluster. The top 10 GOs include neutrophil mediated immunity (GO:0002446), neutrophil degranulation (GO:0043312), neutrophil activation involved in immune response (GO:0002283), antigen processing and presentation of exogenous peptide antigen *via* MHC class I (GO:0042590), antigen processing and presentation of exogenous peptide antigen *via* MHC class I, TAP-dependent (GO:0002479), cytokine-mediated signaling pathway (GO:0019221), aerobic electron transport chain (GO:0019646), mitochondrial ATP synthesis coupled electron transport (GO:0042775), Fc-gamma receptor signaling pathway (Go:0038094), and cellular protein modification process (GO:0006464). **(D)** KEGG of DEGs in NK cluster. The top 10 KEGGs include Parkinson disease, Prion disease, Amyotrophic lateral sclerosis, Pathways of neurodegeneration, Huntington disease, Pathogenic Escherichia infection, Shigellosis, Salmonella infection, Proteasome, and Alzheimer’s disease.

To investigate the IRG expression characteristics of PBMCs in AD, DEGs of each cluster were screened to generate IRGs based on the ImmPort database, which summarized IRGs from published studies, and 212 IRGs were obtained from DEGs of each cluster in PBMCs (**Table S2**). The AUCell R package was used to determine the IRG activity of each cell line (**Figure 3A**). Cells expressing more genes exhibited higher AUC values, and these cells were mainly in NK and DC cells, colored in yellow (**Figure 3B**). As NK clusters were remarkably decreased in the AD group (**Figures 2B, F**), we further performed GO and KEGG analysis of DEGs in the NK cluster from PBMCs. These terms were mainly related to antigen processing and immune response (**Figures 3C, D**).

### DEGs of AD Brain From Bulk Sequencing Data

**Figure 4:**
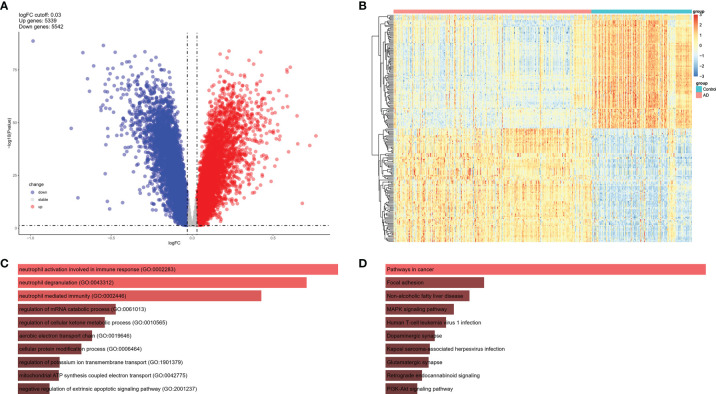
DEGs of AD brain from GSE33000 dataset. **(A)** Volcano plot of DEGs (|logFC| > 0.03 and adjusted P value < 0.05). Up-regulated genes were colored in red and down-regulated genes were colored in blue. **(B)** Heatmap of top 100 up and top 100 down DEGs of GSE33000. **(C)** GO of DEGs in GSE33000. The top 10 GOs include neutrophil activation involved in immune response (GO:0002283), neutrophil degranulation (GO:0043312), neutrophil mediated immunity (GO:0002446), regulation of mRNA catabolic process (GO:0061013), regulation of cellular ketone metabolic process (GO:0010565), aerobic electron transport chain (GO:0019646), cellular protein modification process (GO:0006464), regulation of potassium ion transmembrane transport (GO:1901379), mitochondrial ATP synthesis coupled electron transport (GO:0042775), and negative regulation of extrinsic apoptotic signaling pathway (GO:2001237). **(D)** KEGG of DEGs in GSE33000. The top 10 KEGGs include Pathways in cancer, Focal adhesion, Non-alcoholic fatty liver disease, MAPK signaling pathway, Human T-cell leukemia virus 1 infection, Dopaminergic synapse, Kaposi sarcoma-associated herpesvirus infection, Glutamatergic synapse, Retrograde endocannabinoid signaling, and PI3K-Akt signaling pathway.

To investigate the expression features of brain tissues in AD, the bulk RNA sequencing dataset GSE33000, which included 310 AD patients and 157 controls, was analyzed to explore DEGs in AD brains and screen the common IRGs between PBMCs and brain tissues. DEGs with adjusted P values < 0.05, and |logFC| > 0.03 were selected (**Table S3**). Thereafter, 5,339 up and 5542 down-regulated DEGs were retained in AD brains (**Figure 4A**). A heatmap of the top100 upregulated and top100 downregulated DEGs is shown, and relative consistency was observed within groups (**Figure 4B**). Interestingly, similar to the expression features of the NK cluster in the PBMC of AD, the top 10 GO terms of DEGs in AD brains also focused on immune response (**Figures 4C, D**). These data indicate that DEGs of the NK cluster in AD PBMCs shared similar GO terms with DEGs in AD brains, suggesting a potential role of NK cells in the brain of AD patients.

### Common IRGs and Relevant Regulatory Transcription Factors

**Figure 5:**
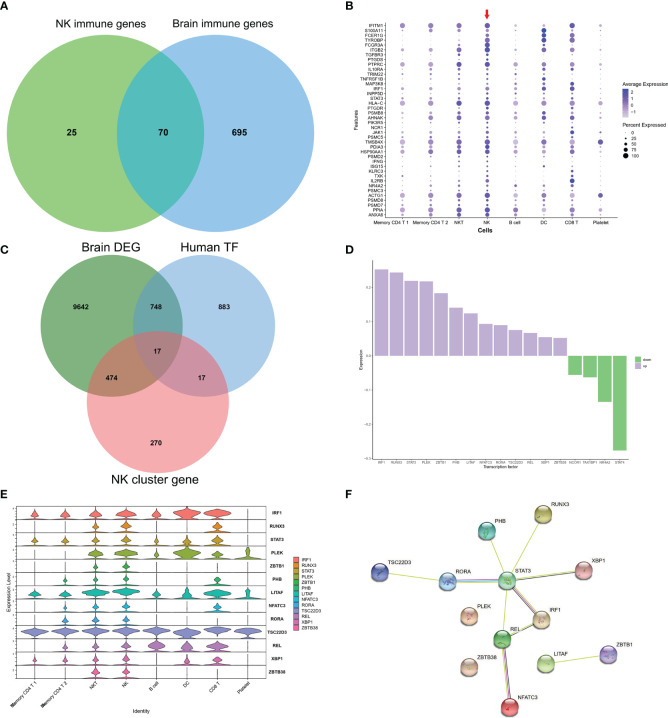
Common IRGs and relevant regulatory transcription factors. **(A)** Venn plot showed IRGs of NK cluster from PBMC in GSE181279 and IRGs in DEGs of AD brains in GES33000. A total of 70 common IRGs were found in both PBMC NK cluster and AD brains. **(B)** Dot plot of top 40 common IRGs in PBMCs. The red arrow dictates NK cluster from PBMC in GSE181279. **(C)** Venn plot showed TFs in NK cluster from PBMC in GSE181279, Human TF database, and TFs in DEG of AD brains in GES33000. **(D)** The expression of common TFs in DEG of GES33000. Up-regulated TFs were colored in purple, and down-regulated TFs were colored in green. **(E)** The expression of common TFs in AD PBMCs. **(F)** The PPI network of the common TFs illustrated using STRING. STAT3 serves as a hub gene.

As GO of DEGs in NK cluster from PBMCs and GO of DEGs from AD brains mainly focused on immune response, we further investigated the common expression characteristics of IRGs between peripheral NK cells and the AD brain. A total of 70 common IRGs were found in both the PBMC NK cluster and AD brains (**Figure 5A** and **Table S4**). Expression of the top 40 common IRG DEGs in GSE33000 was mainly active in the NK cluster from PBMCs (**Figure 5B**).

To investigate the transcriptionally regulated activity of IRGs, a list of 1665 TFs was obtained from HumanTFDB (http://bioinfo.life.hust.edu.cn/HumanTFDB/#!/) (19). 34 TFs in the AD NK cluster from PBMCs and 765 TFs in the AD brain were retained. Furthermore, 17 common TFs were expressed in both the AD peripheral NK cluster and the AD brain (**Figure 5C**). The FindAllMarkers function was applied using default parameters (only.pos = TRUE, min.pct = 0.25, logfc.threshold = 0.25). In this situation, only positive genes expressed in more than 25% of cells were selected with a logFC cutoff of 0.25. A total of 1979 unique marker genes were identified among all clusters. For the NK cluster, a total of 778 marker genes were identified, and only 34 genes belonged to TFs. Therefore, the overlapping TFs among peripheral NK marker genes, DEGs in AD brains, and TF database were only 17. The expression of the 17 TFs in GSE33000 and AD peripheral NK clusters is shown (**Figures 5D, E**). Thirteen TFs were upregulated in the AD brain, and all 17 TFs were active in NK and NKT cells. The PPI network suggests that STAT3 may play a critical role as a hub gene in the transcriptional regulation of IRGs (**Figure 5F**).

### Confirmation of NK Infiltration in AD Brain

**Figure 6:**
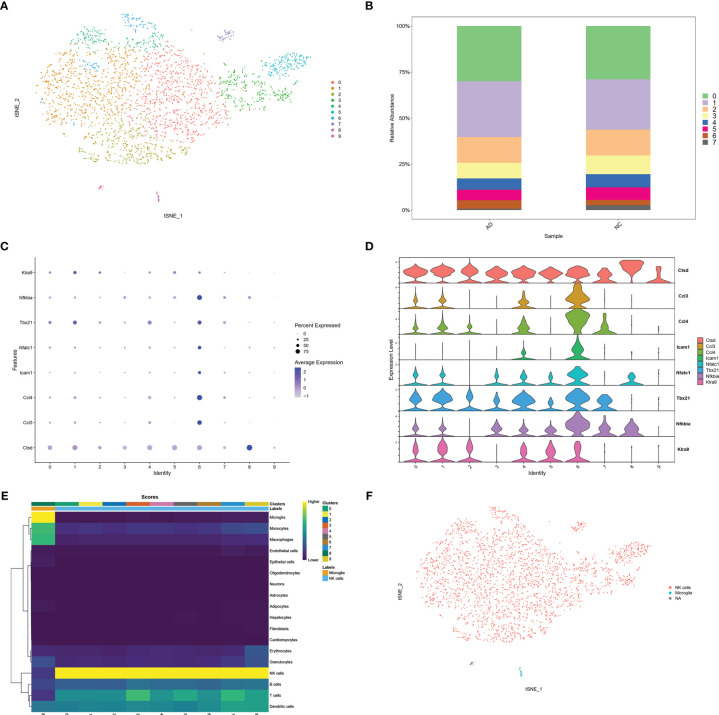
scRNA analysis of NK cells in AD brain. **(A)** tSNE projection of 3,250 cells from all NK cells isolated from brain of AD mice. **(B)** Cluster distribution in each sample. **(C)** Dot plot of NK related genes. **(D)** Violin plot of NK related genes. **(E)** Score plot of cluster annotation by singleR package. **(F)** tSNE projection of annotated clusters.

To confirm infiltration of NK cells into the brain. We selected the scRNA sequencing dataset GSE142853 from the GEO database, which sequenced sorted NK cells from the brains of 3XTg-AD mice. A total of 3,250 cells, including 1,634 cells from AD mice and 1,616 cells from NC mice. Ten clusters were generated (**Figure 6A**). The percentage of cluster 6 was higher in the AD group than in the NC group (4.6% vs. 2.9%) (**Figure 6B**). The cytotoxic molecule (*Cstd*), pro-inflammatory chemokines (*Ccl3, Ccl4*), adhesion molecules (*Icam1*), and NK cell activation molecules (*Tbx21, Nfatc1, Nfkbia, and Klra9*) were highly expressed in cluster 6 (**Figures 6C, D**). Moreover, SingleR and Celldex R packages were used to annotate these clusters, and the prediction accuracy was calculated using scores. SingleR is an automatic annotation method used for scRNA sequencing data (20). SingleR provides a reference expression dataset of samples with known cell type labels, and it can label new cells based on similarity to the reference. The celldex R package provides an easy way to apply the SingleR package for the annotation of scRNA sequencing data. Higher scores represented more accurate predictions, and NK cells were annotated with the highest score. The results showed that 9 of the 10 clusters in GSE142853 were annotated as NK cells, while one cluster was annotated as microglia. Both NK and microglia annotations were labeled in yellow, representing higher accuracy (**Figure 6E**). The tSNE plot showed a very small proportion of microglia and the majority of NK cells in the brains of 3XTg-AD mice (**Figure 6F**). Taken together, the GSE142853 dataset confirmed the occurrence of NK cells in AD brains, and some of the infiltrated circulating NK cells were activated in the AD brain.

## Discussion

Neuroinflammation is a pathological hallmark of AD, and clonally expanded CD8^+^ TEMRA cells have been found in both peripheral mononuclear cells and cerebrospinal fluid (CSF) of AD patients (21). The functional link between the peripheral immune system and the CNS is increasingly recognized. The proteomic landscape of peripheral blood revealed several biomarkers for AD diagnosis, including peripheral inflammatory biomarkers (22–24). In AD, high inflammation, reactive microglia, leaky BBB, buildup of plaques, and NFTs attract infiltration of peripheral immune cells that interact with resident microglia. The CNS and peripheral immune systems interact to contribute to AD (25, 26). CD8 ^+^ T cells infiltrated the brains of APP-PS1AD transgenic mice and regulated the expression of synapse- and neuronal-related genes (27). However, little is known about the infiltration of NK cells into the AD brain.

NK cells are a subgroup of cytotoxic lymphocytes that belong to the innate immune system. NK cells respond and kill cells quickly upon activation without pre-stimulation (28). NK cells release cytotoxic granules to initiate apoptosis in other cells or release inflammatory cytokines, including IFN-γ, to stimulate other immune cells (29). The cytotoxic activity of peripheral NK cells is impaired in aged individuals compared to that in young adults during aging (30). Circulating NK cell infiltration into the brain has been found in human AD and 3xTg-AD mice (6, 7). NK cells exhibited enhanced pro-inflammatory profiles in the brains of 3xTg-AD mice. Depletion of NK cells *via* anti-NK1.1 antibody remarkably reduced neuroinflammation, enhanced neurogenesis, and ameliorated cognitive impairment in 3xTg-AD mice without affecting Aβ concentrations. After depletion of NK cells, microglia show a decreased proliferative response and decreased expression of pro-inflammatory cytokines (7). Targeting NK cells may shed new light to combat AD.

In this study, we used scRNA sequencing data to illustrate the landscape of immune cells and IRG characteristics from PBMCs in AD. A total of eight clusters were identified, and a decreased proportion of NK cells was found in the AD group compared to the NC group (**Figure 2F**). IRGs are essential for immune cells to respond to immune stimulation and immune infiltration (31). An immune gene list including 212 genes was constructed by combining DEGs of each cluster and immune genes from the ImmPort database. The IRG scores were calculated for all cells according to the expression of IRGs, and high scores were mainly found in NK and DC cells (**Figure 3B**). In the bulk RNA sequencing data of the AD human brain, we identified 5,339 upregulated and 5542 downregulated DEGs. The GO analysis of bulk RNA sequencing data was similar to scRNA sequencing data from PBMCs, indicating that the peripheral and CNS showed similar expression characteristics of IRGs in AD.

We further explored the potential regulatory mechanisms of these common IRGs by investigating TF DEGs. A total of 17 common TFs were found in both peripheral NK cells and AD brain tissues, among which 13 TFs were overexpressed. PPI network analysis suggested that STAT3 may be the hub TF involved in the regulation of IRGs. STAT3 is activated in infiltrating immune cells in tumors (32). Blockage of STAT3 is accompanied by decreased NK infiltration and reduced levels of IFN-γ in lymphoma (33). These studies suggest that STAT3 may play a critical role in NK activation and infiltration into the brain in AD.

To confirm NK infiltration in the AD brain, we also analyzed another scRNA sequencing dataset of sorted NKs in the brains of 3XTg-AD mice. The AD mice showed an increased proportion of activated NK cluster (**Figures 6B–D**). The singleR package confirmed the purity of the NK cells (**Figure 6E**). These data further confirmed the infiltration and activation of NK cells in the AD brain.

The BBB is impaired in AD, making infiltration of peripheral cells into the brain possible. The reduction of NK clusters in PBMCs of AD patients raised the speculation of NK infiltration into the brain. In another scRNA dataset of sorted NK cells from AD brains (GSE142853), NK cells were confirmed using cell-specific marker genes. Moreover, several genes representing NK activation were found to be increased in the subgroups of infiltrated NK cells in AD brains. The results from GSE142853 confirmed the infiltration and activation of peripheral NK cells. Taken together, the data from GSE181279 and GSE142853 may indicate that CNS infiltration leads to the reduction of peripheral NK cells. However, further *in vivo* studies are required to validate the CNS infiltration of peripheral NK cells.

In conclusion, we propose that peripheral NK cells may infiltrate the brain and contribute to neuroinflammatory changes in AD using bioinformatic analysis combining both scRNA and bulk sequencing data. Moreover, STAT3 may be involved in the transcriptional regulation of IRGs, infiltration, and activation of NK cells.

## Data Availability Statement

The datasets presented in this study can be found in online repositories. The names of the repository/repositories and accession number(s) can be found in the article/**Supplementary Material**.

## Author contributions

XW and YH conceived and designed the study. YL and KL collected data. XW wrote the paper. All authors contributed to the article and approved the submitted version.

## Funding

This work was supported by grants from the National Natural Science Foundation of China (No. 81801069 to YH, 81901103 for KL, and No. 81500925 to XW).

## Conflict of Interest

The authors declare that the research was conducted in the absence of any commercial or financial relationships that could be construed as a potential conflict of interest.

## Publisher’s Note

All claims expressed in this article are solely those of the authors and do not necessarily represent those of their affiliated organizations, or those of the publisher, the editors and the reviewers. Any product that may be evaluated in this article, or claim that may be made by its manufacturer, is not guaranteed or endorsed by the publisher.

## Abbreviations

AD, Alzheimer’s disease; Aβ, amyloid β; NFTs, neurofibrillary tangles; CNS, central nervous system; BBB, blood-brain barrier; IRGs, Immunity related genes; scRNA, single-cell RNA; PBMCs, peripheral blood mononuclear cells; HVGs, highly variable genes; PCA, principal component analysis; DEGs, differentially expressed genes; AUC, area under the curve; PPI, protein-protein interaction; UMI, unique molecular identifier.
